# Improving Self-Care in Patients With Coexisting Type 2 Diabetes and Hypertension by Technological Surrogate Nursing: Randomized Controlled Trial

**DOI:** 10.2196/16769

**Published:** 2020-03-27

**Authors:** Calvin Kalun Or, Kaifeng Liu, Mike K P So, Bernard Cheung, Loretta Y C Yam, Agnes Tiwari, Yuen Fun Emmy Lau, Tracy Lau, Pui Sze Grace Hui, Hop Chun Cheng, Joseph Tan, Michael Tow Cheung

**Affiliations:** 1 Department of Industrial and Manufacturing Systems Engineering University of Hong Kong Hong Kong Hong Kong; 2 Department of Information Systems, Business Statistics, and Operations Management Hong Kong University of Science and Technology Hong Kong Hong Kong; 3 Department of Medicine University of Hong Kong Hong Kong Hong Kong; 4 Ambulatory Diabetes Centre Pamela Youde Nethersole Eastern Hospital Hong Kong Hong Kong; 5 Jockey Club School of Public Health and Primary Care Chinese University of Hong Kong Hong Kong Hong Kong; 6 School of Nursing Hong Kong Sanatorium and Hospital Hong Kong Hong Kong; 7 Diabetes Mellitus Centre Tung Wah Eastern Hospital Hong Kong Hong Kong; 8 DeGroote School of Business McMaster University Hamilton, ON Canada

**Keywords:** technological surrogate nursing, eHealth, complex chronic disease, diabetes, hypertension, self-care, patient safety

## Abstract

**Background:**

Technological surrogate nursing (TSN) derives from the idea that nurse-caregiver substitutes can be created by technology to support chronic disease self-care.

**Objective:**

This paper begins by arguing that TSN is a useful and viable approach to chronic disease self-care. The analysis then focuses on the empirical research question of testing and demonstrating the effectiveness and safety of prototype TSN supplied to patients with the typical complex chronic disease of coexisting type 2 diabetes and hypertension. At the policy level, it is shown that the data allow for a calibration of TSN technology augmentation, which can be readily applied to health care management.

**Methods:**

A 24-week, parallel-group, randomized controlled trial (RCT) was designed and implemented among diabetic and hypertensive outpatients in two Hong Kong public hospitals. Participants were randomly assigned to an intervention group, supplied with a tablet-based TSN app prototype, or to a conventional self-managing control group. Primary indices—hemoglobin A_1c_, systolic blood pressure, and diastolic blood pressure—and secondary indices were measured at baseline and at 8, 12, 16, and 24 weeks after initiation, after which the data were applied to test TSN effectiveness and safety.

**Results:**

A total of 299 participating patients were randomized to the intervention group (n=151) or the control group (n=148). Statistically significant outcomes that directly indicated TSN effectiveness in terms of hemoglobin _1c_ were found in both groups but not with regard to systolic and diastolic blood pressure. These findings also offered indirect empirical support for TSN safety. Statistically significant comparative changes in these primary indices were not observed between the groups but were suggestive of an operational calibration of TSN technology augmentation. Statistically significant changes in secondary indices were obtained in one or both groups, but not between the groups.

**Conclusions:**

The RCT’s strong behavioral basis, as well as the importance of safety and effectiveness when complex chronic illness is proximately self-managed by layperson patients, prompted the formulation of the empirical joint hypothesis that TSN would improve patient self-care while satisfying the condition of patient self-safety. Statistical and decision analysis applied to the experimental outcomes offered support for this hypothesis. Policy relevance of the research is demonstrated by the derivation of a data-grounded operational calibration of TSN technology augmentation with ready application to health care management.

**Trial Registration:**

ClinicalTrials.gov NCT02799953; https://clinicaltrials.gov/ct2/show/NCT02799953

## Introduction

The management of chronic disease requires dedicated and joint efforts from health care professionals and patients. Current literature has emphasized the importance of empowering the latter in the direction of self-care [1-4]. Individuals with long-term conditions, such as diabetes and hypertension, are required to maintain proper levels of blood glucose (BG) and blood pressure (BP), follow medication instructions, and lead healthy lifestyles. However, continuing challenges over compliance, motivation, and organization have been noted in the literature [5-9]. Technology is increasingly being called upon to assist in such endeavors. In particular, electronic health (eHealth) protocols are suggested to facilitate patient-doctor-nurse communication, mutually informed decision making, access to health care resources, and the organization, interpretation, and dissemination of health data [10-17]. The ready availability of technology on the supply side prompts a question on the demand side: Would such interventions be accepted and used by chronically ill patients to the extent of significantly improving self-care? Unless this question is resolved endogenously and the success factors explicated, chronic disease management could turn out to be a costly and unrewarding task.

Earlier studies of technological support in chronic disease self-management have focused on single health conditions and have reported mixed results [18-21]. Following de Boer et al [22], we recognize that chronic disease is generally complex, with the coexistence of type 2 diabetes and hypertension being the *common case*. Additionally, it is recognized that individuals who are ill in this way would be best served by care that is given at just the right time, is available all the time, is up to date, and given with a response that is friendly and immediate. As resource limitations render this humanly impracticable, it can be asked whether substitutes produced by technology would encourage and empower chronically ill patients to self-provide the desired care. In answer, we submit that technology would be able to create substitute nurse-caregivers, under the umbrella designation of technological surrogate nursing (TSN). TSN would effectively and safely support self-care on the part of chronically ill patients at the levels of attention, immediacy, and timeliness determined by medical, engineering, and economic considerations. Since TSN can be supplied to meet a wide range of specifications, it can be deemed to be typical among technology-based interventions dedicated to self-care.

In this paper, research supporting our thesis is approached on an empirical level. As noted above, where self-care is required for chronic illnesses, the *common case* is professionally regarded to be that of coexisting type 2 diabetes and hypertension. The first efforts under our empirical research question are, therefore, directed toward testing the effectiveness of TSN when applied to self-care under the conditions of representative chronic disease and representative technology. Safety is submitted to be an equally important requirement in the self-management of complex chronic diseases, where health care responsibility is shared between health care professionals and patients, but proximate action is undertaken by the patients with limited and asymmetric layperson knowledge and medical expertise. Taking the above considerations into account, a randomized controlled trial (RCT) was designed and applied to parallel groups of patients with both diabetes and hypertension to enable the empirical analysis of the effectiveness along with the safety of a prototype eHealth TSN. The two fundamental requirements, together with the experiment’s strong behavioral basis, prompted the formulation of the joint hypothesis that TSN would improve patient self-care while satisfying the condition of patient self-safety. In the following sections, our experiment and its outcomes are presented and interpreted and empirical support offered for this joint hypothesis. In addition, policy relevance is demonstrated by the derivation from the data of an operational calibration of TSN technology augmentation with ready application to health care management, such as cost-benefit analysis.

The exposition is organized along the lines of the research question of this paper, in particular with regard to experimental methodology and outcomes interpretation and analysis.

## Methods

### Trial Design and Participants

We designed and implemented a 24-week, parallel-group RCT to test TSN effectiveness and safety among patients with coexisting type 2 diabetes and hypertension. Participants in the experiment were recruited from two diabetes outpatient clinics of two public hospitals in Hong Kong. The individuals were aged 18 years and over; had received a physician-confirmed diagnosis of type 2 diabetes and hypertension at least one month prior; were prescribed oral medication in consequence; were, by declaration, able to self-manage chronic conditions; and were able to read Chinese and speak Cantonese. Excluded from the study were individuals with visual, cognitive, or physical impairments or with unstable or life-threatening illnesses that precluded self-management.

The study was approved by the Hong Kong East Cluster Research Ethics Committee (reference: HKEC-2015-058). All participants provided written informed consent.

### Recruitment and Follow-Up

Outpatients in the diabetes clinics were invited to an information session, where the trial was introduced and eligibility determined. Selected individuals were then visited at home, where the research protocols were explained, eligibility confirmed, and written informed consent obtained. Baseline BP and BG were measured under seated conditions after 5 minutes of sitting rest. BP measurements were made twice, 1 minute apart, by automated 2-in-1 BG and BP monitors; averages of the two readings were computed as examination values. BG levels in terms of hemoglobin A_1c_ (HbA_1c_) were measured by applying point-of-care HbA_1c_ analyzers. These data were applied to subsequent stratification and analysis. A questionnaire was administered to collect demographic and other health-related information. Participants were randomized to an intervention group (IG) or a control group (CG) as described in the following subsection. Follow-up home visits were made at 8, 12, 16, and 24 weeks postrandomization. HbA_1c_ was measured at 12 and 24 weeks, while BP and other health-related data were recorded at 8, 16, and 24 weeks.

### Randomization and Masking

Participants were stratified into four groups according to baseline HbA_1c_ and systolic BP (SBP): (1) HbA_1c_ ≤8.0% and SBP ≤159 mmHg, (2) HbA_1c_ ≤8.0% and SBP ≥160 mmHg, (3) HbA_1c_ >8.0% and SBP ≤159 mmHg, and (4) HbA_1c_ >8.0% and SBP ≥160 mmHg. Parameter values were based on previous research [23,24]. Individuals in each subgroup were randomized by means of randomly permuted blocks of four and six with sequentially numbered, sealed opaque envelopes, all of which were implemented centrally by a research team member over the telephone. Participants and researchers were all blinded to the randomization; however, given the nature of the intervention, blinding was not possible postrandomization.

### Prototype Technological Surrogate Nursing and Other Experimental Equipment

Participants randomized to the IG were supplied with prototype eHealth TSN developed by the research team with reference to clinician inputs, needs, and expectations in chronic disease self-care identified in usability evaluations [25,26] and other literature [27,28], as well as funding constraints. Running on a tablet computer, this protocol offered, within limits of its design and with due attention to safety, timely and interactive access to procedures and resources dedicated to the self-management of type 2 diabetes and hypertension. In particular, measurement and recording of BG and BP were enabled through Bluetooth-connected BG and BP monitors. These data, presented in structured tables and charts, were readily retrievable and reviewable. Authorized outside individuals (eg, caregivers and family members) were allowed access to the TSN platform through a secure Web portal. A module was available to provide text- and video-based learning resources pertaining to the causes and prevention of type 2 diabetes and hypertension, self-care, exercise, diet, health plans, and stress management. An audio function was programmable to emit action reminders at predetermined times. CG participants were supplied with BG and BP monitors of the same type, with logbooks as specified under conventional self-management protocols. Individuals in both groups were trained and encouraged to apply the tools in question to self-monitor and self-record BG and BP, and to adhere to other self-care activities recommended by primary care providers.

### Outcome Measures

TSN effectiveness was measured, firstly, by indices representing the fundamental clinical manifestations under complex diabetes and hypertension: BG, as indicated by HbA_1c_, and BP, as indicated by SBP and diastolic BP (DBP) [23,24]. TSN effectiveness, at a level once-removed from the clinical level, was measured in terms of the following secondary indices:

1. Medication adherence, defined by the frequency of failure to follow medication during the past 2 months and evaluated by five items adapted from the Morisky Medication Adherence Scale [29]: 1 (never), 2 (rarely), 3 (sometimes), 4 (always), and 5 (all the time). Item scores were reversed during data analysis to allow higher scores to correspond to greater adherence.
2. General adherence to treatment, defined by whether the individual followed prescribed treatments during the past 2 months and evaluated by the five-item Medical Outcomes Study General Adherence Scale [30]: 1 (none of the time), 2 (a little of the time), 3 (some of the time), 4 (a good bit of the time), 5 (most of the time), and 6 (all of the time).
3. Adherence to disease-specific activities, defined by the frequency of performance of disease-specific activities during the past 2 months and evaluated using the 15-item Medical Outcomes Study Disease-Specific Adherence Scale [30]: 1 (none of the time), 2 (a little of the time), 3 (some of the time), 4 (a good bit of the time), 5 (most of the time), and 6 (all of the time).
4. Diabetes knowledge and hypertension knowledge assessed in terms of responses to 11 and 25 true or false questions, respectively [31].
5. Self-efficacy for coping with chronic disease, defined as confidence in performing self-management and assessed using five items adapted from a validated scale [32], with scores ranging from 1 (not at all confident) to 10 (totally confident).

Frequencies of self-monitoring of BG and BP were recorded as the number of times per week. As shown in the Discussion section, a method is suggested under which the primary and secondary indices would indirectly indicate the presence or absence of patient self-safety.

### Statistical Analysis

According to a priori power calculations based on an assumed 10% loss to follow-up, a sample of 147 individuals per group would provide over 80% power (two-sided alpha=.05) to detect a between-group mean difference of 5 mmHg for SBP (SD 14.5), 4 mmHg for DBP (SD 10), and 0.5% for HbA_1c_ (SD 1.25). Independent *t* tests and chi-square tests were applied to compare differences in baseline characteristics between the IG and the CG members. We applied both intention-to-treat (without imputation) analysis and per-protocol analysis, under linear mixed-modelling of mean changes in indices within and between groups. Effects were classified according to time (ie, follow-ups versus baseline), treatment group (ie, IG versus CG), and group-by-time interaction. Self-monitoring frequency was compared between the two groups using the Mann-Whitney U test. Sensitivity analysis was introduced after adjusting for baseline variables.

## Results

### Study Characteristics

**Figure 1:**
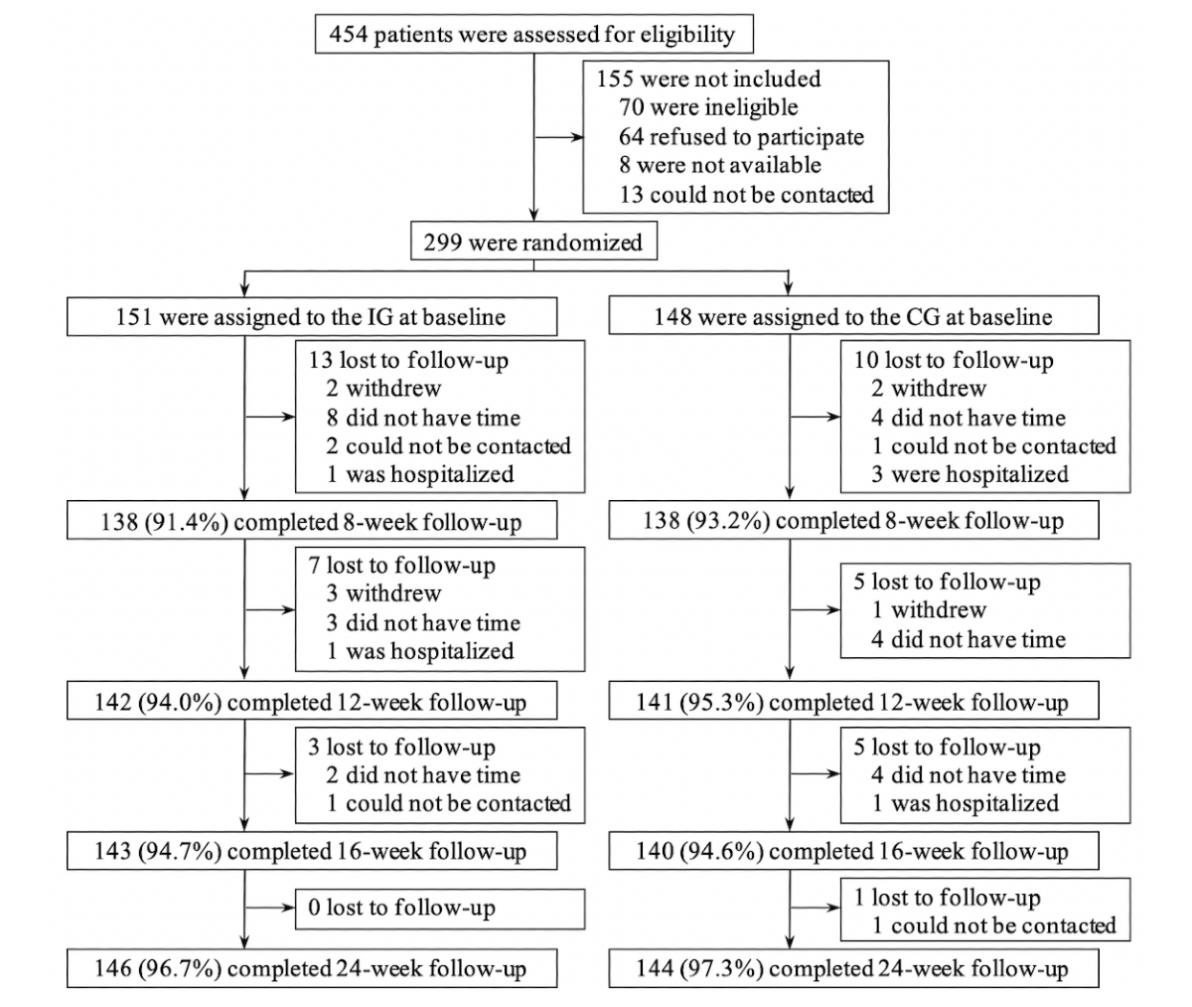
Trial flowchart. CG: control group; IG: intervention group.

Between March and October 2016, 151 patients were randomized to the IG and 148 patients were randomized to the CG (see Figure 1 for the trial flowchart). As shown in Table 1, there were no statistically significant differences between the groups with regard to baseline characteristics, except for experience in the use of computer-based self-monitoring systems (*P*=.02).

**Table 1:** Baseline characteristics by study group.

Characteristic		Intervention group (n=151)	Control group (n=148)	*P* value
Age (years), mean (SD)		63.9 (10.2)	63.7 (9.6)	.90
**Sex, n (%)**				**.09**
	Male	104 (68.9)	88 (59.5)	
	Female	47 (31.1)	60 (40.5)	
**Education, n (%)**				**.09**
	No schooling completed	0 (0)	4 (2.7)	
	Some primary school	10 (6.6)	14 (9.4)	
	Completed primary school	25 (16.6)	21 (14.2)	
	Some secondary school	23 (15.2)	23 (15.5)	
	Completed secondary school	66 (43.7)	47 (31.8)	
	Diploma, advanced diploma, associate degree, or the equivalent	10 (6.6)	21 (14.2)	
	Bachelor’s degree	14 (9.3)	12 (8.1)	
	Master’s degree	3 (2.0)	5 (3.4)	
	Doctoral degree	0 (0)	1 (0.7)	
**Habitation status, n (%)**				**.61**
	Living alone	21 (13.9)	15 (10.1)	
	Living with family	129 (85.4)	132 (89.2)	
	Other	1 (0.7)	1 (0.7)	
Duration of diabetes (years), mean (SD)		15.6 (10.0)	16.6 (11.3)	.42
Duration of hypertension (years), mean (SD)		12.9 (8.7)	12.9 (9.9)	>.99
**Experience using computers, tablets, or mobile phones, n (%)**				**.85**
	Yes	117 (77.5)	116 (78.4)	
	No	34 (22.5)	32 (21.6)	
Time spent using a computer, tablet, or mobile phone (hours per day), mean (SD)		2.4 (3.1)	3.2 (4.1)	.06
**Experience using computer-based self-management systems, n (%)**				**.02**
	Yes	3 (2.0)	12 (8.1)	
	No	148 (98.0)	136 (91.9)	
**Experience with self-management support programs and training, n (%)**				**.23**
	Yes	100 (66.2)	88 (59.5)	
	No	51 (33.8)	60 (40.5)	
Hemoglobin _1c_ (%), mean (SD)		8.02 (1.54)	7.99 (1.23)	.88
Systolic blood pressure (mmHg), mean (SD)		137.6 (17.7)	137.4 (15.4)	.92
Diastolic blood pressure (mmHg), mean (SD)		76.2 (9.8)	74.7 (10.3)	.20

### Intention-to-Treat Analysis

#### Primary Outcomes

**Figure 2:**
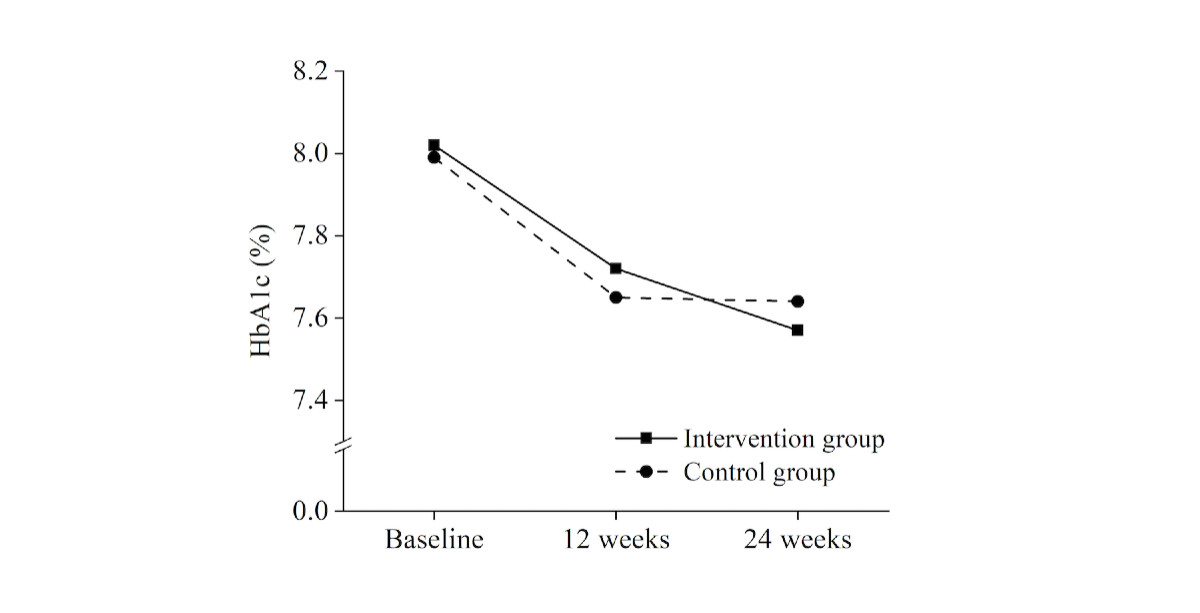
Mean hemoglobin A_1c_ (HbA_1c_) levels during the 24-week study period.

**Figure 3:**
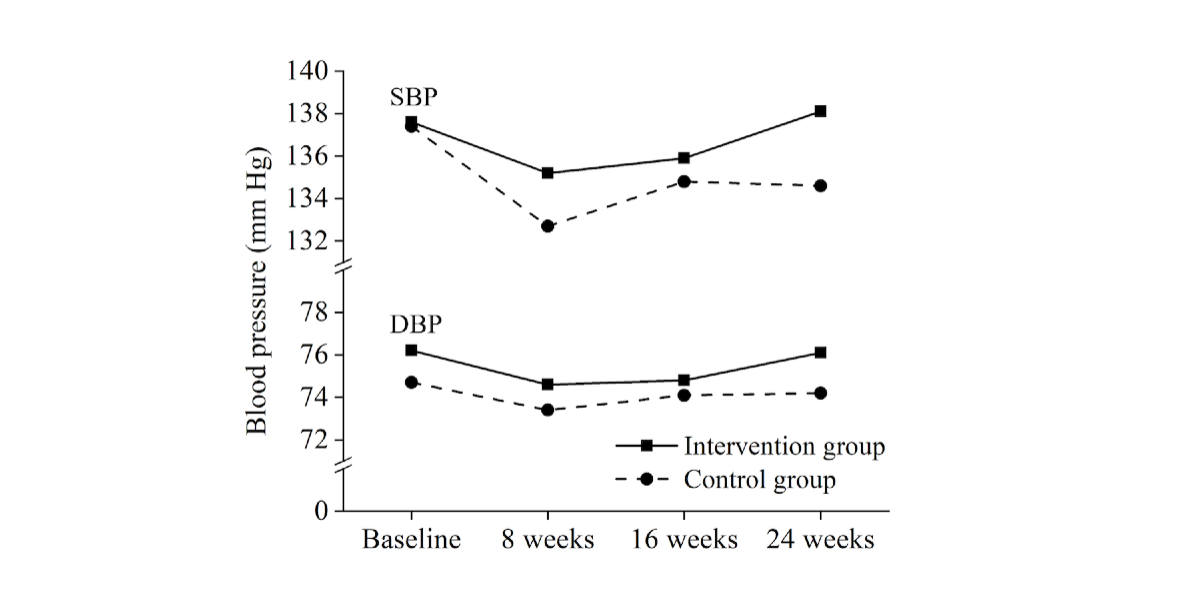
Mean systolic blood pressure (SBP) and diastolic blood pressure (DBP) during the 24-week study period.

Outcomes with direct reference to TSN effectiveness are displayed in Table 2. Mean changes in HbA_1c_ were found to be statistically significant at all assessment times in both the IG and the CG (see Figure 2). Comparing HbA_1c_ mean changes between the two groups, statistically significant outcomes were not observed. With regard to SBP and DBP, mean changes in each group were mixed in statistical significance (see Figure 3). Differences in SBP and DBP mean changes between the IG and the CG were not statistically significant. With regard to TSN patient safety, indirect implications of the above findings will be pursued below in the Discussion section.

**Table 2:** Results of intention-to-treat analysis.

Outcomes			Intervention group (n=151), mean (95% CI)		Control group (n=148), mean (95% CI)		Between-group difference with regard to change in outcome from baseline (95% CI)		*P* value	
**Primary outcomes**										
	**Hemoglobin A_1c_ (%)**									
		Baseline		8.02 (7.80-8.23)		7.99 (7.77-8.21)		N/A^a^		
		12 weeks		7.72 (7.50-7.94)^b^		7.65 (7.43-7.87)^b^		0.05 (−0.23 to 0.33)		.74
		24 weeks		7.57 (7.35-7.79)^b^		7.64 (7.42-7.86)^b^		−0.09 (−0.37 to 0.19)		.52
	**Systolic blood pressure (mmHg)**									
		Baseline		137.6 (134.8-140.3)		137.4 (134.6-140.2)		N/A		
		8 weeks		135.2 (132.3-138.0)		132.7 (129.9-135.6)^b^		2.25 (−1.87 to 6.37)		.28
		16 weeks		135.9 (133.0-138.7)		134.8 (132.0-137.7)		0.84 (−3.26 to 4.93)		.69
		24 weeks		138.1 (135.3-140.9)		134.6 (131.7-137.4)		3.38 (−0.68 to 7.44)		.10
	**Diastolic blood pressure (mmHg)**									
		Baseline		76.2 (74.6-77.7)		74.7 (73.1-76.2)		N/A		
		8 weeks		74.6 (73.0-76.2)^b^		73.4 (71.8-75.0)		−0.31 (−2.26 to 1.64)		.76
		16 weeks		74.8 (73.3-76.4)		74.1 (72.5-75.7)		−0.80 (−2.74 to 1.14)		.42
		24 weeks		76.1 (74.5-77.6)		74.2 (72.6-75.8)		0.33 (−1.59 to 2.25)		.73
**Secondary outcomes**										
	**Medication adherence (score)**									
		Baseline		4.52 (4.45-4.59)		4.53 (4.46-4.60)		N/A		
		8 weeks		4.64 (4.57-4.71)^b^		4.57 (4.50-4.64)		0.08 (−0.01 to 0.16)		.07
		16 weeks		4.58 (4.51-4.65)^b^		4.53 (4.46-4.60)		0.06 (−0.02 to 0.14)		.14
		24 weeks		4.58 (4.51-4.65)^b^		4.56 (4.49-4.63)		0.03 (−0.05 to 0.12)		.40
	**General adherence to treatment (score)**									
		Baseline		4.17 (4.03-4.32)		4.00 (3.86-4.15)		N/A		
		8 weeks		4.15 (4.00-4.30)		3.95 (3.80-4.10)		0.03 (−0.16 to 0.22)		.78
		16 weeks		4.17 (4.02-4.32)		4.03 (3.88-4.18)		−0.03 (−0.22 to 0.16)		.75
		24 weeks		4.27 (4.12-4.42)		3.98 (3.83-4.13)		0.12 (−0.07 to 0.31)		.21
	**Adherence to disease-specific activities (score)**									
		Baseline		3.51 (3.42-3.61)		3.51 (3.41-3.61)		N/A		
		8 weeks		3.55 (3.45-3.65)		3.65 (3.55-3.75)^b^		−0.10 (−0.22 to 0.03)		.12
		16 weeks		3.73 (3.63-3.83)^b^		3.66 (3.56-3.76)^b^		0.06 (−0.06 to 0.18)		.35
		24 weeks		3.72 (3.62-3.82)^b^		3.63 (3.53-3.73)^b^		0.08 (−0.04 to 0.20)		.19
	**Diabetes knowledge (%)**									
		Baseline		78.5 (76.7-80.3)		79.1 (77.2-80.9)		N/A		
		8 weeks		81.5 (79.7-83.4)^b^		82.6 (80.7-84.5)^b^		−0.53 (−3.21 to 2.15)		.70
		16 weeks		84.2 (82.4-86.1)^b^		84.1 (82.2-86.0)^b^		0.65 (−2.01 to 3.31)		.63
		24 weeks		84.4 (82.6-86.3)^b^		85.4 (83.5-87.2)^b^		−0.40 (−3.04 to 2.24)		.77
	**Hypertension knowledge (%)**									
		Baseline		72.4 (70.7-74.2)		70.9 (69.1-72.6)		N/A		
		8 weeks		73.2 (71.4-75.0)		73.6 (71.7-75.4)^b^		−1.95 (−4.38 to 0.47)		.11
		16 weeks		75.6 (73.8-77.4)^b^		74.9 (73.1-76.7)^b^		−0.82 (−3.22 to 1.59)		.50
		24 weeks		76.7 (74.9-78.5)^b^		76.2 (74.4-78.0)^b^		−1.01 (−3.40 to 1.38)		.41
	**Self-efficacy for coping with chronic disease (score)**									
		Baseline		7.31 (7.09-7.53)		6.98 (6.75-7.21)		N/A		
		8 weeks		7.38 (7.15-7.61)		7.17 (6.94-7.40)		−0.12 (−0.39 to 0.15)		.38
		16 weeks		7.45 (7.22-7.68)		7.24 (7.02-7.47)^b^		−0.12 (−0.39 to 0.14)		.36
		24 weeks		7.49 (7.26-7.72)		7.24 (7.02-7.47)^b^		−0.08 (−0.35 to 0.18)		.55

^a^N/A: not applicable.

^b^Significant difference from baseline.

#### Secondary Outcomes

As shown in Table 2, statistically significant direct improvements in TSN effectiveness as measured by adherence to disease-specific activities, diabetes knowledge, and hypertension knowledge were observed in both the IG and the CG at 16 and 24 weeks. Mixed results were obtained for the shorter times. Improvements in medication adherence were significant in the IG at all follow-up points, and improvements in self-efficacy for coping with chronic diseases were significant for the CG at 16 and 24 weeks. Outcomes for general adherence to treatment were all statistically nonsignificant. Significant differences in the secondary indices were not observed between the groups at any follow-up points. Indirect implications of the above findings for TSN patient safety will be pursued in the Discussion section.

### Per-Protocol and Sensitivity Analyses

Per-protocol analysis showed that in the IG, the statistically nonsignificant intention-to-treat outcomes for DBP (at 16 weeks) and for self-efficacy in coping with chronic disease (at 24 weeks) became significant, with the opposite change applying to medication adherence at 16 weeks and 24 weeks (see Multimedia Appendix 1). Sensitivity analysis showed that the results were unchanged after adjusting for baseline variables, with the exception that in the CG, the statistically nonsignificant change in self-efficacy in coping with chronic disease at 8 weeks became significant (see Multimedia Appendix 2).

### Self-Monitoring Frequency

Over the 24-week study period, participants in both groups displayed similar mean frequencies (times per week) with regard to self-monitoring of BG (IG mean 4.67, SD 4.45, median 3.65; CG mean 4.47, SD 3.67, median 3.62; *P*=.94) and with regard to self-monitoring of BP (IG mean 5.26, SD 6.03, median 3.46; CG mean 4.07, SD 3.54, median 3.17; *P*=.16).

## Discussion

### Principal Findings

Statistically significant outcomes that directly indicate TSN effectiveness in terms of HbA_1c_ were found for both the IG and the CG, but not with regard to SBP and DBP. Significant comparative changes in the three primary indices were not observed between the groups. Significant changes in secondary indices were obtained in one or both groups, but not between the groups.

### Interpretation of Individual Outcomes

In line with its empirical orientation, this study is focused on measurement, outcome interpretation, and hypothesis testing. On the analytical level, it is sufficient for such purposes that the data were derived from the impact of TSN-supported or conventional self-management on diabetic and hypertensive patient behavior, which in turn was structured under the experimental design and manifested empirically by way of the primary and secondary indices. With regard to the interpretation of outcomes, the RCT’s strong behavioral basis prompts a reference to the axiom of rationality in economics and decision science. Under this axiom, individual behavior and changes therein are required to not consistently inflict self-harm [33]. In health care, the idea of *no harm* is embedded in a *sine qua non*, which requires that any treatment or situations must be safe for the individuals concerned. A similar *sine qua non* in the aviation industry is that of passenger safety.

The requirement of *no harm* is especially important in the self-management of complex chronic illnesses, such as diabetes and hypertension, where health care professionals and patients share the health care responsibility but proximate action is undertaken by patients with asymmetric and limited layperson knowledge of the interdependent medical manifestations and effects. In view of this action and knowledge asymmetry, *no harm* can be operationally interpreted to represent the minimum level of safety perceived to be acceptable by the rational patient with regard to their own health care−related activities. As this would be the case with every individual involved in every self-managed health care situation, *no harm* can be formalized linguistically to mean the lower bound of self-safety. Mathematically, *no harm* can, therefore, be understood to be the denoting description of the minimal element in a set of self-managed health situations ordered by the perception-based asymmetric binary relation *≤*, read from right to left as “(perceived to be) more than or equally safe.”

To facilitate the interpretation of outcomes in terms of the health care *sine qua non*, a short mathematical discussion is presented [34]. The concepts introduced above can be operationalized in terms of a numerical mapping to represent the total or partial ordering *≤*. A number of technical problems then arise, as follows: the existence of many levels of self-safety leads to the questions of defining its maximum commensurability between factors entering such a definition; the possibility that an upper limit cannot be meaningfully delineated so that, in defiance of practical experience, the set of health situations must be left open ended (ie, specified to be of countably infinite cardinality); differentiation between *maximum safety* and neighboring *very safe* situations; and the order-intransitivity that emerges when this distinction is not clearly established. Until problems of this nature are resolved, the domain of self-managed health situations would be incompletely defined and partially ordered. Numerical representation of the self-safety relation *≤* would require continuous real mapping over such a domain, which additionally must be monotonic and unique under linear transformation in any completely defined and ordered subdomain. The specification of a probability function and a random variable to represent patient self-safety under such circumstances would involve additional applications of measure theory.

Under the study’s empirical approach, these analytical difficulties can be mitigated by careful experimental design and data analysis. Reference to the formal description presented above suggests that the *sine qua non* of safety can be satisfied in the strong form, which involves the condition of at least *no harm*, or in the weak form, which involves the condition of *no harm*. To operationalize and test the empirical existence of the *sine qua non* in the case of chronic disease self-management, where the outcomes have an important dependence on layperson knowledge and action, it would be sufficient to demonstrate that its weak form requiring the singleton instance of *no harm*, or patient-perceived minimum safety, is supported by the data. The measurement problem would then be considerably less demanding than if the strong form of the *sine qua non* is involved; witness the case of health care quality assurance, where accommodation of the strong form would lead to incompletely resolved problems of cardinal measurement and numerical comparability of incremental safety over many situations [35].

An additional dimension to the measurement problem arises in the case of complex chronic diseases, such as diabetes and hypertension (ie, that illness is generally manifested in more than one form). Numerical representation of patient self-safety would, therefore, require reference to different health manifestations under a given complex chronic disease (eg, BG and BP in the case of diabetes and hypertension). As shown in the Methods section, BG and BP manifestations can properly be measured in terms of HbA_1c_, SBP, and DBP indices. Instead of adding complexity to the numerical representation of self-safety, availability of these index numbers suggests a method to reduce the difficulties by carefully interpreting the experimental design and experimental data.

To ease the exposition, we first present three supporting observations. First, because an index number is a mapping function from a set of data to a set of real numbers, the suggested method exploits the mathematical property that under a function, each element in its range—an index value in this case—is the image of a distinct element in the domain—a data point in this case. Second, since index functions are operational and, hence, mathematically constructible, it is possible to focus attention on one computed point in its range—an index value—and one point in its domain—a data point—so that a 1:1 relationship would follow *ipso facto*. Third, it was pointed out earlier that difficult analytical problems must be resolved before a random variable can be constructed to characterize self-safety under sampling. Though statistical tests applicable to such conditions are not yet available, a second-best approach can properly be sought to extract from the data the wherewithal to indicate whether the health care *sine qua non* is present in weak form.

A second-best method to exploit index number statistical results to indicate minimum patient self-safety can be conveniently explained by example. As reported in Table 2, results involving the HbA_1c_ index were statistically significant in the IG at all assessment dates. TSN can then be concluded to have impacted positively on patient self-care. Positive impact on patient well-being follows immediately, especially as it has been shown that lowering HbA_1c_ by 1% can translate to as much as a 40% reduction in the risk of microvascular complications and a 20% reduction in the risk of diabetes-related deaths [36]. Individuals reaping health benefits of this nature can properly be deemed to be at least unharmed; therefore, with regard to TSN, minimum patient self-safety would be assured upon indirect interpretation of the HbA_1c_ index results. Similar reasoning would apply to the other statistically significant index number outcomes in the IG (see Table 2) (ie, DBP for 8 weeks; medication adherence for 8, 16, and 24 weeks; adherence to disease-specific activities for 16 and 24 weeks; diabetes knowledge for 8, 16, and 24 weeks; and hypertension knowledge for 16 and 24 weeks). Referring to the IG outcome for SBP at 24 weeks, it is suggested that since the incremental average in question was measured to be numerically positive and, hence, clinically negative, nonsignificance would instead become the statistical property of concern. Given that this was indeed found to be the case so that statistically significant evidence of self-harm was not forthcoming, it is proper to indirectly infer that TSN was accompanied by minimum patient self-safety in this instance.

The remaining index number outcomes in the IG, though statistically nonsignificant (see Table 2), are amenable to *empirically necessary reasoning* under the second-best method. To explain by example, we first refer to the CG data to draw an inference regarding patient self-safety on the following methodological grounds. Given that self-care was practiced among the CG members following established health care procedures, it can be assumed *ipso facto* that, independent of effectiveness, the resulting outcomes would be at least not harmful and, hence, at least minimally safe. Average index data from the group can, therefore, be properly assumed to have been produced under the auspices of patient self-management characterized by at least *no harm*; hence, they can be sifted for necessary indications of minimum self-safety even in the absence of formal statistical analysis. Notice that the *consistent* condition of the underlying rationality axiom referred to above is accommodated by averaging in the index measurements.

Recall the idea, which was broached above, that index numbers measuring health manifestations such as BP can be exploited to reduce difficulties when measuring patient safety. Consider then how the SBP and DBP primary indices would perform under the suggested data gleaning—as shown earlier, HbA_1c_ has been pre-empted into the group of indices appropriate for indirect interpretation. From Table 2, it is seen that in the CG, DBP at 8, 16, and 24 weeks showed hypertension improvements before statistical testing, so that the incremental index averages (ie, 1.26, 0.53, and 0.44, respectively) expressed clinically positive effects at these dates. By virtue of the fact that only CG data entered into these computations, at least *no harm*—and *a fortiori* *no harm—*can be properly assumed to have accompanied the self-care that produced the effects in question. Combining these observations and the mathematical properties of functions noted earlier, it is argued that numerical representation of minimum patient self-safety can be attributed to the DBP index when applied to CG average incremental data. This is true in the sense that such a computation would provide the basis for a clinically meaningful index value to be assigned in order to functionally image a data point known to have been produced from a *no harm* situation. By implication, under the 1:1 linguistic relationship, this index value would also numerically represent the denoting minimum self-safety itself. It is thus possible, under this kind of *empirically necessary reasoning*, to interpret the DBP reading of 1.26 mmHg to be the numerical representation at 8 weeks of the minimum self-safety denoting a *no harm* situation embedded in the CG data at the same time. Similar reasoning suggests that the other DBP index values of 0.53 and 0.44 in the CG can be interpreted to numerically represent minimum self-safety at 16 weeks and 24 weeks, respectively. Repeating the argument, the SBP incremental index values of 4.65, 2.54, and 2.81 in Table 2 can be understood to functionally image *no harm* data points for the CG and, hence, to numerically represent the denoting minimum self-safety at 8, 16, and 24 weeks, respectively.

Empirically necessary indications of minimum patient self-safety obtained on the basis of CG data can be carried over to the IG by reference to the between-group comparative findings in Table 2; they can also be carried over by exploiting the fact that the individuals involved were originally part of a single cohort satisfying the same basic health criteria (see the Methods section). Given this shared origin and the random nature of patient assignment to the IG and the CG, behavior in the two groups can properly be assumed to derive from similar perceptions regarding safety when self-managing diabetes and hypertension. Returning to the result that differences in SBP and DBP outcomes between the groups were statistically nonsignificant and focusing on the clinically positive cases, we can now interpret the index averages in question to be empirically equivalent, pairwise. It is further noted that between the two groups, individual indices can be matched 1:1 with assessment date as the parameter. In Table 2, it is therefore seen that clinically positive incremental index averages for DBP at 8, 16, and 24 weeks in the CG can be matched with empirically equivalent, clinically positive incremental index averages for DBP at 8, 16, and 24 weeks in the IG. Understanding this exercise in terms of empirically necessary reasoning, indications of minimum patient self-safety obtained in the CG are thus carried over to the IG. Repeating the argument would show that clinically positive incremental index averages for SBP at 8 and 16 weeks in the CG can be matched with empirically equivalent clinically positive incremental index averages for SBP at 8 and 16 weeks in the IG, so a carryover can again be made. The 24-week IG reading for SBP was found to be clinically negative, so it cannot be matched with the clinically positive 24-week SBP reading in CG. Instead, as already shown, it was interpreted indirectly. 

Therefore, it is observed that a second-best method, based on index numbers and applying indirect statistical interpretation or empirically necessary reasoning, can be found to extract support for the presence of minimum self-safety from the experimental data, even when formal statistical tests are not available. To the extent that the secondary index outcomes satisfy the method’s conditions, it would again apply. Similar to the previous argument, findings of statistically nonsignificant differences in secondary outcomes between the IG and the CG (see Table 2) would allow clinically positive incremental index averages to be identified and matched using assessment date as the parameter. It is seen from Table 2 that minimum self-safety would then be indicated in the cases of medication adherence, adherence to disease-specific activities, diabetes knowledge, hypertension knowledge, and self-efficacy for coping with chronic disease. Application to the remaining secondary outcome of general adherence to treatment is methodologically excluded.

General adherence to treatment covers a wider scope than the other secondary indices, which are all focused on diabetes and hypertension. The activities in question—dieting, exercise, and weight control—are important to self-management under other chronic conditions that may afflict the participants under study (eg, smoking, obesity, and chronic obstructive pulmonary disease) and that may be differently supported by technology or not at all. Asynchronous progress in these directions would impact the patient’s diabetic and hypertensive status in a distributed lag, which cannot be meaningfully compared with the single-dated effects measured under the other secondary indices. Therefore, it is proposed that until the research is properly extended, general adherence to treatment should be excluded from outcomes interpretation involving the other secondary indices, on methodological grounds of differences in scope, behavioral basis, and *ceteris paribus* specifications.

Further interpretation of the BP-based outcomes is suggested if a maintained hypothesis implicit in the experimental design is recalled, that additional knowledge is a necessary condition for consistent changes in behavior. Referring to the statistically nonsignificant SBP and DBP outcomes and the statistically significant knowledge-related outcomes in Table 2, an anomaly would seem to emerge, which is that individuals learned more about hypertension and yet significant impact on BP control did not follow. A resolution is suggested by noting that because of diminishing returns on hypertension treatment [37], small changes in BP such as the ones observed are likely in the case of long-term patients. We also note the view that it is not difficult for the layperson to understand the rudimentary aspects of hypertension [38]. It is then additionally likely that the long-term patients entering each group already possessed working knowledge of hypertension. It follows that with little addition to hypertension knowledge, the behavior of long-term patients would be little affected, to the extent of producing statistically insignificant changes in already-low SBP and DBP measurements.

### Effectiveness Along With Patient Safety Under Technological Surrogate Nursing

It was noted in the previous section that empirical analysis of TSN effectiveness and TSN self-safety should properly proceed in parallel. Under the health care *sine qua non*, the first question asked in practice is whether *no harm* would hold in an absolute sense inside the case with minimum reference to outside situations. This suggests that the real-world evaluation of TSN effectiveness should also be approached from an absolute perspective, under which the RCT outcomes are interpreted in the capacity of empirical analysis subject to strong other-things-being-equal conditions. This procedure is in line with common scientific practice, under which research is generally evaluated along two headings depending on *ceteris paribus* conditions: absolutely or relatively. Safety is similarly important as effectiveness in chronic disease self-management due to layperson-patient involvement. In addition, the experimental data were measured in terms of independent indices under clinically delineated conditions; hence, they are, mensuration-wise, consistent with the absoluteness required by the health care *sine qua non*. Consequently, it is methodologically permissible for TSN self-safety to formally appear together with TSN effectiveness to be empirically tested under a joint hypothesis. We, therefore, propose the following: other things being equal, TSN would improve self-care among chronically ill diabetic and hypertensive patients while satisfying the *sine qua non* of patient self-safety.

It has been shown that 13 statistically significant primary and secondary outcomes supported the empirical existence of direct positive incremental impact of TSN on patient self-care and indirect positive incremental impact on patient self-safety. In addition, 10 primary and secondary outcomes offered empirically necessary indications of accompanying minimum patient self-safety. It was shown that minimum patient self-safety yielded sufficient empirical support for the health care *sine qua non*. It was also shown that the first (ie, absolute outcomes evaluation) heading noted above covered the primary indices HbA_1c_ (2 cases) and DBP (1 case), as well as the secondary indices medication adherence (3 cases), adherence to disease-specific activities (2 cases), diabetes knowledge (3 cases), and hypertension knowledge (2 cases). In addition, it was shown that the outcomes under the second (ie, relative outcomes evaluation) heading covered the primary indices SBP (3 cases) and DBP (2 cases), as well as the secondary indices adherence to disease-specific activities (1 case), hypertension knowledge (1 case), and self-efficacy for coping with chronic disease (3 cases). This listing is seen to be exhaustive, given that the number of possible outcomes forthcoming in the IG sum to the same total of 23 (see Table 2), with one secondary index being excluded on methodological grounds. Therefore, it is submitted that the IG data offer statistically significant and empirically necessary support to the joint hypothesis presented above.

### Comparative Findings and Calibration of Technological Surrogate Nursing Technology Augmentation

Under the second scientific approach of relative evaluation, the key statistical finding is that, as compared between the IG and the CG, there was no difference in effectiveness of self-care (see Table 2). Self-safety was demonstrated to be empirically equivalent between the groups and so would play a neutral role in the present argument. As a matter of research methodology, a null result of this nature can properly claim a place in the literature [39]. As discussed in the Methods section, since dedicated technological support was absent in the RCT’s CG, it is further suggested that the result’s empirical implications are not exhausted but can be usefully pursued under the *quaesitum* of adding technology to self-care. If the RCT is interpreted as a *thought experiment*, we can understand the comparative data to reflect the *as-if* effects of adding technology to diabetes and hypertension self-management, which, up to then, did not make use of it. Thought experiments and as-if reasoning, which were famously exploited by Einstein, would be familiar to physicists [40]. Health care is generally asymmetrical, as witnessed by the near-impossibility of recreating the patient’s initial state by reversing treatment. In this case, however, individuals receiving prototype TSN improved in self-care and, yet, individuals without it were not worse off. The apparent anomaly is resolved under the thought experiment by suggesting that the technology in question is so elementary that adding it to standard (ie, control) practice did not produce empirically significant effects. The following question then emerges: Would further addition of technology to the TSN prototype (ie, technology augmentation) increase effectiveness?

Analysis under this question can proceed operationally with support from the comparative data. First, it is suggested that the prototype technology represents the lowest meaningful level in TSN design. The IG data were, therefore, obtained under such a technological condition. Second, we recall the seminal Michelson-Morley experiment and its consequences for calibration in physics [40]. An experimentally grounded base level is, therefore, proposed by analogy, with reference to which technology augmentation in TSN can be operationally calibrated in two dimensions: its degree in any given case being measured by engineering index comparison with the base technology, and its effectiveness being measured against base-outcomes indices. For example, a flexible internet-based, tethered or mobile, TSN platform with artificial intelligence (AI) capabilities can be determined to be m-times more advanced than the base (ie, prototype) version; this is in terms of an index of information technology and computational requirements and its effectiveness measured incrementally under an index covering the base outcomes. Applying the calibration over a properly chosen range of cases would yield a numerical representation of TSN technology augmentation in terms of a ranking of technology and a ranking of effectiveness. Since expert knowledge can be relied on to supply engineering indices for incremental comparison, the first ranking is cardinal and monotonic with origin determined by base technology. Given that the second ranking is constructed from incremental magnitudes, it is naturally cardinal with origin determined by base effectiveness. However, since patient acceptance is involved, this ranking may or may not be monotonic; it is possible for individuals to feel overloaded with technology, in which case incrementally negative effects leading to local nonmonotonicity would emerge.

The suggested calibration is operational, simple (ie, only two constructs), and empirically grounded. It can readily be introduced into health care management, such as TSN cost-benefit analysis. Following the principle of bounded rationality [33], decision complexity would be reduced by eliminating nonmeaningful alternatives through the extraction of a monotonic subordering from the effectiveness ranking; decision complexity would be further reduced by delineation of benefits with reference to this subordering and of costs with reference to the parallel-technology ranking. The solution to the cost-benefit analysis would then determine optimal TSN and optimal choice of technology; since the latter may or may not be determined to be at the most advanced level, the question of whether introducing more technology into health care would be rational in an already technology-loaded society can be resolved under clearly specified conditions.

### Suggestions for Paradigm Development

The data suggest a potentially important implication for the treatment of complex chronic disease and, hence, for TSN paradigm development: the emergence of differential effects. It is noted that in each experimental group, the same individuals receiving the same medication and the same self-care were involved when measuring HbA_1c_, SBP, and DBP. Changes in these indices can, therefore, be validly compared within the group. Note, the influence of baseline values is excluded under incremental measurement. If the patterns revealed by pairwise comparisons in each group (see Table 2)—falling HbA_1c_ against little change in SBP and falling HbA_1c_ against little change in DBP—are interpreted in terms of health manifestations under complex chronic diabetes and hypertension, it is suggested that patients relatively more afflicted with diabetes would be more responsive to self-management than patients suffering relatively more from hypertension. Though the observation is *chartist*, it suggests the empirical existence of differential effects. If formal statistical evidence is forthcoming, empirical grounds follow to justify paradigm development to extend TSN to selectable multitasking under an umbrella of complex chronic disease (eg, obesity, heart failure, chronic obstructive pulmonary diseases, and chronic skeletal-joint problems, in addition to diabetes and hypertension), so as to encourage technology-induced behavioral change along the lines of greater patient response.

The increasing and increasingly successful applications of AI to health care point to a potentially fruitful direction for TSN paradigm development [41-44]. Referring to the classic cybernetic observation that the individual represents a spatially and temporally local pocket of decreasing entropy (ie, increasing organization), and that disease impacts negatively on this status [45], the *human-machine team* [46] of diabetic and hypertensive patients and prototype TSN presented in this paper can be interpreted to be an eHealth system constructed to slow down the latter process. Formally, this system can be imagined to contain a second-order, cybernetic, human subsystem in communication and interaction with a first-order, cybernetic, machine subsystem. The eHealth systems dedicated to different patients would be embedded in a larger system constructed to manage complex chronic diabetes and hypertension.

A suggestion for paradigm development follows immediately, under which the prototype TSN would be upgraded to a second-order, cybernetic subsystem by the introduction of more advanced monitoring and feedback mechanisms; more importantly, the TSN would be upgraded by the introduction of the AI necessary to converse, as well as interact, with its chronically ill human partner, to remember and analyze the results, to learn, and to persuade in an intelligent and friendly manner. The incremental information and knowledge acquired thereby can then be applied to enhance TSN user experience and safety, increase compliance, and induce behavioral change toward more ordered (ie, lower entropy) and healthier living on the part of the patient [47,48]. In particular, deep-learning AI can first be exploited to search the available health and social data and help construct a TSN protocol to mimic the perceptions and behaviors of a typical individual with chronic diabetes and hypertension. This protocol would be integrated with the TSN platform’s caregiving and patient-safety functions and would be connected to the relevant cloud-based databases to ensure continuous updating and enrichment. With the addition of AI-supported speech capabilities, the enhanced TSN would be readily able to converse with chronically ill individuals and establish empathy while supporting self-management. A feedback channel can then be created and maintained to assist in the provision of immediate self-care guidance and initiate learning on the part of the TSN to enable future guidance and to change the patient’s behavior and goals toward healthier living. In comparison with the prototype technology’s *third-party* audio reminder function, which is described in the Prototype Technological Surrogate Nursing and Other Experimental Equipment subsection, an individual with irregular insulin administration habits would be more inclined to accept admonishment, persuasion, and corrective guidance from a “fellow diabetic” AI-enhanced TSN.

The above endeavors in paradigm development would fall, in part, under the ambit of technology augmentation. Given the absence of AI elements in the prototype technology and, hence, consistent applicability of base-level measurements to AI-based technology augmentation, the calibration presented in the previous section can be introduced to assist cost-benefit analysis in order to determine the best TSN in such cases. In addition to evaluating AI-enhanced TSN human-machine teams in the standard directions, the decision maker can seek to apply the cybernetic condition of team optimality [49] to guard against failure of value alignment between the subsystems [46].

Of interest to the business side is an approach to paradigm development suggested by consumer and especially patient acceptance of internet technology [50-54]. Paradigm development based on technology augmentation and its commercialization involve costly and difficult-to-reverse investment; therefore, it can be asked whether—by free-riding on this kind of consumer acceptance and with only nominal and, hence, readily reversible commitment to new technology—TSN can quickly and cheaply gain demand-side impact among chronically ill individuals. Such a question would be of interest to the health care business manager, such as suggesting informal paradigm development focused on marketing, benefits from free-riding on user experience with “old” but still-extant technology, and the capturing of economic rent implicit in knowledge-based external effects. Consider the following example: since shopping and banking are as much a part of everyday life as health care, consumer acceptance of internet e-shopping and e-banking [50,55] can be exploited in a marketing exercise of this type. By free-riding on well-established experience and familiarity with Technology Acceptance Model characteristics, such as usefulness, ease of use, and reliability, TSN can be advertised in these directions under health-oriented descriptions at lower cost-benefit ratios as compared to a *de novo* effort. Advertisements can suggest that TSN is easily programmable to unfailingly remind the chronically ill patient to take medication on time and in correct dosages. Additional free-riding would be possible by coupling TSN at behaviorally aware junctures to e-shopping or e-banking. For example, AI-generated friendly reminders to “watch the diet” can appear on monitors of the devices used by chronically ill individuals to access e-shops or e-banks. In this way, familiarity of use in one direction would breed familiarity of use and, hence, more patient compliance in the other.

### Study Limitations

This study has several limitations. First, individuals recruited to the RCT were long-term patients with stably controlled conditions, on average. Therefore, our results may not be generalizable to higher-risk cases. Second, for reasons due to funding, the follow-up period was relatively short for the impact of TSN on chronic disease self-management to be more fully revealed. Finally, due to confidentiality concerns on the part of some patients, data regarding drug usage before and after the study were not collected.

### Conclusions

Our research offers empirical support to the joint hypothesis that TSN would improve patient self-care while satisfying the *sine qua non* of patient self-safety. Practical utility is demonstrated by the derivation of an operational calibration of technology augmentation with applicability to health care management such as cost-benefit analysis. On a broader level, our findings suggest increased and wider use of technological surrogates in chronic disease management and help clarify the important question of whether introducing more technology in health care would be rational in an already technology-loaded society. Paradigm development in TSN is especially proposed with regard to multitasked technology augmentation aimed at inducing behavioral change along the lines of greater patient response in increasingly complex disease environments, AI enhancement, and synergy with internet technology and innovation.

## Appendix

Multimedia Appendix 1 Results from per-protocol analyses.

Multimedia Appendix 2 Results from sensitivity analyses.

Multimedia Appendix 3 CONSORT-eHEALTH checklist (V 1.6.1).

## Abbreviations

AI: artificial intelligence
BG: blood glucose
BP: blood pressure
CG: control group
DBP: diastolic blood pressure
eHealth: electronic health
HbA_1c_: hemoglobin A_1c_
IG: intervention group
RCT: randomized controlled trial
SBP: systolic blood pressure
TSN: technological surrogate nursing

## References

[1] Anderson RM, Funnell MM, Butler PM, Arnold MS, Fitzgerald JT, Feste CC (1995). Patient empowerment: Results of a randomized controlled trial. Diabetes Care.

[2] Funnell MM, Anderson RM (2004). Empowerment and self-management of diabetes. Clin Diabetes.

[3] Hernandez-Tejada MA, Campbell JA, Walker RJ, Smalls BL, Davis KS, Egede LE (2012). Diabetes empowerment, medication adherence and self-care behaviors in adults with type 2 diabetes. Diabetes Technol Ther.

[4] (2012). World Health Organization (WHO) Regional Office for Europe.

[5] Bergenstal R, Pearson J, Cembrowski GS, Bina D, Davidson J, List S (2000). Identifying variables associated with inaccurate self-monitoring of blood glucose: Proposed guidelines to improve accuracy. Diabetes Educ.

[6] Moen A, Brennan PF (2005). Health@Home: The work of health information management in the household (HIMH): Implications for consumer health informatics (CHI) innovations. J Am Med Inform Assoc.

[7] Cramer JA (2004). A systematic review of adherence with medications for diabetes. Diabetes Care.

[8] Krousel-Wood M, Joyce C, Holt E, Muntner P, Webber LS, Morisky DE, Frohlich ED, Re RN (2011). Predictors of decline in medication adherence: Results from the cohort study of medication adherence among older adults. Hypertension.

[9] Vervloet M, Linn AJ, van Weert JC, de Bakker DH, Bouvy ML, van Dijk L (2012). The effectiveness of interventions using electronic reminders to improve adherence to chronic medication: A systematic review of the literature. J Am Med Inform Assoc.

[10] Wildevuur SE, Simonse LW (2015). Information and communication technology-enabled person-centered care for the "big five" chronic conditions: Scoping review. J Med Internet Res.

[11] Schooley B, San Nicolas-Rocca T, Burkhard R (2015). Patient-provider communications in outpatient clinic settings: A clinic-based evaluation of mobile device and multimedia mediated communications for patient education. JMIR Mhealth Uhealth.

[12] Lin C, Wittevrongel L, Moore L, Beaty BL, Ross SE (2005). An internet-based patient-provider communication system: Randomized controlled trial. J Med Internet Res.

[13] Hsu J, Huang J, Fung V, Robertson N, Jimison H, Frankel R (2005). Health information technology and physician-patient interactions: Impact of computers on communication during outpatient primary care visits. J Am Med Inform Assoc.

[14] Liederman EM, Morefield CS (2003). Web messaging: A new tool for patient-physician communication. J Am Med Inform Assoc.

[15] Simon SR, Evans JS, Benjamin A, Delano D, Bates DW (2009). Patients' attitudes toward electronic health information exchange: Qualitative study. J Med Internet Res.

[16] Woods SS, Schwartz E, Tuepker A, Press NA, Nazi KM, Turvey CL, Nichol WP (2013). Patient experiences with full electronic access to health records and clinical notes through the My HealtheVet Personal Health Record Pilot: Qualitative study. J Med Internet Res.

[17] Xie Z, Nacioglu A, Or C (2018). Prevalence, demographic correlates, and perceived impacts of mobile health app use amongst Chinese adults: Cross-sectional survey study. JMIR Mhealth Uhealth.

[18] Hou C, Carter B, Hewitt J, Francisa T, Mayor S (2016). Do mobile phone applications improve glycemic control (HbA_1c_) in the self-management of diabetes? A systematic review, meta-analysis, and GRADE of 14 randomized trials. Diabetes Care.

[19] McLean G, Band R, Saunderson K, Hanlon P, Murray E, Little P, McManus RJ, Yardley L, Mair FS, DIPSS co-investigators (2016). Digital interventions to promote self-management in adults with hypertension systematic review and meta-analysis. J Hypertens.

[20] Omboni S, Gazzola T, Carabelli G, Parati G (2013). Clinical usefulness and cost effectiveness of home blood pressure telemonitoring: Meta-analysis of randomized controlled studies. J Hypertens.

[21] Or CK, Tao D (2014). Does the use of consumer health information technology improve outcomes in the patient self-management of diabetes? A meta-analysis and narrative review of randomized controlled trials. Int J Med Inform.

[22] de Boer IH, Bangalore S, Benetos A, Davis AM, Michos ED, Muntner P, Rossing P, Zoungas S, Bakris G (2017). Diabetes and hypertension: A position statement by the American Diabetes Association. Diabetes Care.

[23] Green BB, Cook AJ, Ralston JD, Fishman PA, Catz SL, Carlson J, Carrell D, Tyll L, Larson EB, Thompson RS (2008). Effectiveness of home blood pressure monitoring, Web communication, and pharmacist care on hypertension control: A randomized controlled trial. JAMA.

[24] McMahon GT, Gomes HE, Hickson Hohne S, Hu TM, Levine BA, Conlin PR (2005). Web-based care management in patients with poorly controlled diabetes. Diabetes Care.

[25] Or C, Tao D (2012). Usability study of a computer-based self-management system for older adults with chronic diseases. JMIR Res Protoc.

[26] Tao D, Or C (2012). A paper prototype usability study of a chronic disease self-management system for older adults. Proceedings of the IEEE International Conference on Industrial Engineering and Engineering Management.

[27] Murray E, Hekler EB, Andersson G, Collins LM, Doherty A, Hollis C, Rivera DE, West R, Wyatt JC (2016). Evaluating digital health interventions: Key questions and approaches. Am J Prev Med.

[28] Or CKL, Valdez R, Casper G, Carayon P, Burke LJ, Brennan PF, Karsh B (2009). Human factors and ergonomics in home care: Current concerns and future considerations for health information technology. Work.

[29] Morisky DE, Green LW, Levine DM (1986). Concurrent and predictive validity of a self-reported measure of medication adherence. Med Care.

[30] Kravitz R, Hays RD, Sherbourne CD, DiMatteo MR, Rogers WH, Ordway L, Greenfield S (1993). Recall of recommendations and adherence to advice among patients with chronic medical conditions. Arch Intern Med.

[31] Gazmararian JA, Williams MV, Peel J, Baker DW (2003). Health literacy and knowledge of chronic disease. Patient Educ Couns.

[32] Lorig KR, Ritter P, Stewart AL, Sobel DS, Brown BW, Bandura A, Gonzalez VM, Laurent DD, Holman HR (2001). Chronic disease self-management program: 2-year health status and health care utilization outcomes. Med Care.

[33] Simon HA (1979). Rational decision making in business organizations. Am Econ Rev.

[34] Simmons GF (1963). Introduction to topology and modern analysis.

[35] (2019). Agency for Healthcare Research and Quality.

[36] Stratton IM, Adler AI, Neil HA, Matthews DR, Manley SE, Cull CA, Hadden D, Turner RC, Holman RR (2000). Association of glycaemia with macrovascular and microvascular complications of type 2 diabetes (UKPDS 35): Prospective observational study. BMJ.

[37] Mills KT, Bundy JD, Kelly TN, Reed JE, Kearney PM, Reynolds K, Chen J, He J (2016). Global disparities of hypertension prevalence and control: A systematic analysis of population-based studies from 90 countries. Circulation.

[38] Oliveria SA, Chen RS, McCarthy BD, Davis CC, Hill MN (2005). Hypertension knowledge, awareness, and attitudes in a hypertensive population. J Gen Intern Med.

[39] Brassington I (2017). The ethics of reporting all the results of clinical trials. Br Med Bull.

[40] Einstein A, Infeld L (1938). The Evolution of Physics: The Growth of Ideas From Early Concepts to Relativity and Quanta.

[41] Topol E (2019). Deep Medicine: How Artificial Intelligence Can Make Healthcare Human Again.

[42] Meskó B (2019). The real era of the art of medicine begins with artificial intelligence. J Med Internet Res.

[43] Triantafyllidis AK, Tsanas A (2019). Applications of machine learning in real-life digital health interventions: Review of the literature. J Med Internet Res.

[44] Lovis C (2019). Unlocking the power of artificial intelligence and big data in medicine. J Med Internet Res.

[45] Wiener N (1988). The Human Use of Human Beings: Cybernetics and Society.

[46] Russell S (2019). Human Compatible: Artificial Intelligence and the Problem of Control.

[47] Martelaro N, Ju W (2018). Cybernetics and the design of the user experience of AI systems. Interactions.

[48] Kocaballi AB, Berkovsky S, Quiroz JC, Laranjo L, Tong HL, Rezazadegan D, Briatore A, Coiera E (2019). The personalization of conversational agents in health care: Systematic review. J Med Internet Res.

[49] Lai KK, Cheung MT, Fu Y (2017). Resource allocation in public healthcare: A team-DEA model. J Syst Sci Complex.

[50] Liao Z, Cheung MT (2002). Internet-based e-banking and consumer attitudes: An empirical study. Inf Manage.

[51] Or CKL, Karsh B (2009). A systematic review of patient acceptance of consumer health information technology. J Am Med Inform Assoc.

[52] Or CKL, Karsh B, Severtson DJ, Burke LJ, Brown RL, Brennan PF (2011). Factors affecting home care patients' acceptance of a Web-based interactive self-management technology. J Am Med Inform Assoc.

[53] Yan M, Or C (2018). Factors in the 4-week acceptance of a computer-based, chronic disease self-monitoring system in patients with type 2 diabetes mellitus and/or hypertension. Telemed J E Health.

[54] Yan M, Or C (2019). A 12-week pilot study of acceptance of a computer-based chronic disease self-monitoring system among patients with type 2 diabetes mellitus and/or hypertension. Health Informatics J.

[55] Liao Z, Cheung MT (2001). Internet-based e-shopping and consumer attitudes: An empirical study. Inf Manage.

