# Supplementary material for: Improving Self-Care in Patients With Coexisting Type 2 Diabetes and Hypertension by Technological Surrogate Nursing: Randomized Controlled Trial
Source: J Med Internet Res. 2020 Mar 27;22(3):e16769. doi: 10.2196/16769 (PMC7148548; doi:10.2196/16769)
Supplement: Multimedia Appendix 1 [file jmir_v22i3e16769_app1.docx]

Appendix Table 1. Results from per-protocol analysis. Means with 95% confidence intervals, between-group differences with regards to the change in outcomes from baseline, and probabilities.

|  | Mean (95% confidence interval) | | Between-group difference with regards to the change in outcome from baseline (95% confidence interval), and corresponding *P* value |
| --- | --- | --- | --- |
|  | Intervention group (IG)  (n=134) | Control group (CG)  (n=132) |  |
| **Primary outcomes** | | | |
| Hemoglobin A1c (%) (IG: n=142; CG: n=140) | | | |
| Baseline | 7.99 (7.77, 8.22) | 7.98 (7.75, 8.20) | – |
| 12 weeks | 7.71 (7.49, 7.94)* | 7.64 (7.42, 7.87)* | 0.05 (-0.23, 0.33), .72 |
| 24 weeks | 7.56 (7.34, 7.79)* | 7.63 (7.40, 7.85)* | -0.08 (-0.36, 0.20), .57 |
| Systolic blood pressure (mmHg) | | | |
| Baseline | 137.7 (134.8, 140.6) | 137.5 (134.6, 140.4) | – |
| 8 weeks | 135.2 (132.3, 138.1) | 134.1 (131.2, 137.0)* | 0.87 (-3.24, 4.98), .68 |
| 16 weeks | 135.5 (132.6, 138.4) | 135.2 (132.2, 138.1) | 0.15 (-3.96, 4.27), .94 |
| 24 weeks | 138.0 (135.1, 140.9) | 134.8 (131.8, 137.7) | 3.04 (-1.07, 7.15), .15 |
| Diastolic blood pressure (mmHg) | | | |
| Baseline | 76.1 (74.5, 77.8) | 74.6 (73.0, 76.3) | – |
| 8 weeks | 74.5 (72.9, 76.2)* | 73.5 (71.9, 75.2) | -0.48 (-2.50, 1.54), .64 |
| 16 weeks | 74.7 (73.0, 76.3)* | 74.1 (72.4, 75.7) | -0.92 (-2.95, 1.10), .37 |
| 24 weeks | 76.0 (74.3, 77.6) | 74.2 (72.6, 75.9) | 0.24 (-1.78, 2.26), .82 |
| **Secondary outcomes** | | | |
| Medication adherence | | | |
| Baseline | 4.53 (4.46, 4.60) | 4.56 (4.49, 4.63) | – |
| 8 weeks | 4.63 (4.56, 4.70)* | 4.60 (4.52, 4.67) | 0.07 (-0.02, 0.15), .14 |
| 16 weeks | 4.57 (4.50, 4.64) | 4.55 (4.48, 4.62) | 0.05 (-0.03, 0.14), .22 |
| 24 weeks | 4.58 (4.51, 4.65) | 4.57 (4.50, 4.64) | 0.04 (-0.04, 0.13), .32 |
| General adherence to treatment | | | |
| Baseline | 4.19 (4.04, 4.35) | 4.03 (3.87, 4.18) | – |
| 8 weeks | 4.14 (3.99, 4.30) | 3.97 (3.81, 4.12) | 0.01 (-0.19, 0.21), .91 |
| 16 weeks | 4.15 (3.99, 4.30) | 4.05 (3.89, 4.20) | -0.06 (-0.26, 0.14), .54 |
| 24 weeks | 4.27 (4.12, 4.43) | 3.98 (3.82, 4.14) | 0.13 (-0.07, 0.32), .20 |
| Adherence to disease-specific activities | | | |
| Baseline | 3.53 (3.42, 3.64) | 3.50 (3.39, 3.61) | – |
| 8 weeks | 3.56 (3.46, 3.67) | 3.66 (3.55, 3.77)* | -0.13 (-0.25, 0.002), .054 |
| 16 weeks | 3.74 (3.64, 3.85)* | 3.66 (3.56, 3.77)* | 0.05 (-0.08, 0.18), .45 |
| 24 weeks | 3.73 (3.63, 3.84)* | 3.62 (3.51, 3.73)* | 0.08 (-0.04, 0.21), .20 |
| Diabetes knowledge (%) | | | |
| Baseline | 76.7 (74.7, 78.7) | 77.6 (75.6, 79.6) | – |
| 8 weeks | 79.9 (77.9, 81.8)* | 80.8 (78.8, 82.7)* | -0.05 (-2.82, 2.72), .97 |
| 16 weeks | 82.6 (80.6, 84.6)* | 82.5 (80.5, 84.5)* | 0.97 (-1.80, 3.74), .49 |
| 24 weeks | 82.7 (80.7, 84.7)* | 83.5 (81.5, 85.5)* | 0.06 (-2.71, 2.83), .97 |
| Hypertension knowledge (%) | | | |
| Baseline | 73.1 (71.2, 75.0) | 71.1 (69.2, 73.0) | – |
| 8 weeks | 74.0 (72.1, 75.8) | 73.4 (71.5, 75.3)* | -1.53 (-3.99, 0.94), .22 |
| 16 weeks | 75.7 (73.8, 77.6)* | 75.3 (73.4, 77.2)* | -1.71 (-4.17, 0.76), .18 |
| 24 weeks | 76.6 (74.8, 78.5)* | 76.0 (74.1, 77.9)* | -1.42 (-3.88, 1.05), .26 |
| Self-efficacy for coping with chronic disease | | | |
| Baseline | 7.32 (7.08, 7.56) | 6.98 (6.74, 7.22) | – |
| 8 weeks | 7.41 (7.17, 7.65) | 7.15 (6.91, 7.39) | -0.08 (-0.36, 0.20), .57 |
| 16 weeks | 7.48 (7.24, 7.72) | 7.23 (6.99, 7.48)* | -0.09 (-0.37, 0.18), .50 |
| 24 weeks | 7.53 (7.29, 7.77)* | 7.20 (6.96, 7.44)* | -0.01 (-0.29, 0.26), .93 |

*Indicates significant difference from baseline.
