# Supplementary material for: Improving Self-Care in Patients With Coexisting Type 2 Diabetes and Hypertension by Technological Surrogate Nursing: Randomized Controlled Trial
Source: J Med Internet Res. 2020 Mar 27;22(3):e16769. doi: 10.2196/16769 (PMC7148548; doi:10.2196/16769)
Supplement: Multimedia Appendix 2 [file jmir_v22i3e16769_app2.docx]

Appendix Table 2. Results from sensitivity analyses. Means with 95% confidence intervals, between-group differences from the baseline variable-adjusted comparisons of primary and secondary outcomes between the two groups over 24 weeks, and probabilities.

|  | Mean (95% confidence interval) | | Between-group difference with regards to the change in outcome from baseline (95% confidence interval), and corresponding *P* value | |
| --- | --- | --- | --- | --- |
|  | Intervention group | Control group |  |  |
| **Primary outcomes** | | | | |
| Hemoglobin A1c (%) | | | | |
| Baseline | 8.06 (7.88, 8.24) | 7.96 (7.78, 8.14) | – | |
| 12 weeks | 7.75 (7.57, 7.94)* | 7.62 (7.44, 7.80)* | 0.03 (-0.25, 0.31), .84 | |
| 24 weeks | 7.62 (7.44, 7.80)* | 7.60 (7.42, 7.78)* | -0.09 (-0.37, 0.19), .53 | |
| Systolic blood pressure (mmHg) | | | | |
| Baseline | 137.4 (135.2, 139.6) | 137.5 (135.3, 139.8) | – | |
| 8 weeks | 134.9 (132.6, 137.3) | 132.9 (130.5, 135.2)* | 2.16 (-1.94, 6.26), .30 | |
| 16 weeks | 135.7 (133.4, 138.0) | 135.0 (132.7, 137.4) | 0.81 (-3.27, 4.89), .70 | |
| 24 weeks | 138.0 (135.7, 140.3) | 134.7 (132.4, 137.0) | 3.36 (-0.68, 7.41), .10 | |
| Diastolic blood pressure (mmHg) | | | | |
| Baseline | 75.6 (74.6, 76.7) | 75.2 (74.1, 76.2) | – | |
| 8 weeks | 74.0 (72.9, 75.1)* | 73.9 (72.8, 75.0) | -0.32 (-2.25, 1.62), .75 | |
| 16 weeks | 74.3 (73.2, 75.4) | 74.7 (73.6, 75.8) | -0.84 (-2.76, 1.09), .39 | |
| 24 weeks | 75.5 (74.4, 76.5) | 74.8 (73.7, 75.8) | 0.28 (-1.63, 2.19), .77 | |
| **Secondary outcomes** | | | | |
| Medication adherence | | | | |
| Baseline | 4.52 (4.46, 4.59) | 4.53 (4.46, 4.60) | – | |
| 8 weeks | 4.64 (4.57, 4.71)* | 4.57 (4.50, 4.64) | 0.08 (-0.01, 0.16), .07 | |
| 16 weeks | 4.58 (4.51, 4.65)* | 4.53 (4.46, 4.60) | 0.06 (-0.02, 0.14), .14 | |
| 24 weeks | 4.58 (4.52, 4.65)* | 4.56 (4.49, 4.63) | 0.03 (-0.05, 0.12), .40 | |
| General adherence to treatment | | | | |
| Baseline | 4.18 (4.04, 4.32) | 3.99 (3.85, 4.13) | – | |
| 8 weeks | 4.16 (4.01, 4.30) | 3.95 (3.80, 4.09) | 0.02 (-0.17, 0.21), .80 | |
| 16 weeks | 4.18 (4.03, 4.32) | 4.02 (3.88, 4.17) | -0.03 (-0.22, 0.15), .73 | |
| 24 weeks | 4.28 (4.14, 4.42) | 3.97 (3.83, 4.11) | 0.12 (-0.07, 0.31), .21 | |
| Adherence to disease-specific activities | | | | |
| Baseline | 3.53 (3.43, 3.63) | 3.50 (3.40, 3.59) | – | |
| 8 weeks | 3.57 (3.47, 3.67) | 3.63 (3.53, 3.73)* | -0.10 (-0.22, 0.03), .12 | |
| 16 weeks | 3.74 (3.64, 3.84)* | 3.65 (3.55, 3.75)* | 0.06 (-0.06, 0.18), .34 | |
| 24 weeks | 3.74 (3.64, 3.83)* | 3.62 (3.52, 3.72)* | 0.08 (-0.04, 0.20), .18 | |
| Diabetes knowledge (%) | | | | |
| Baseline | 78.8 (77.0, 80.6) | 78.8 (77.0, 80.6) | – | |
| 8 weeks | 81.8 (80.0, 83.7)* | 82.4 (80.5, 84.2)* | -0.55 (-3.23, 2.13), .69 | |
| 16 weeks | 84.5 (82.7, 86.3)* | 83.9 (82.0, 85.7)* | 0.64 (-2.02, 3.30), .64 | |
| 24 weeks | 84.7 (82.9, 86.5)* | 85.1 (83.3, 87.0)* | -0.43 (-3.07, 2.21), .75 | |
| Hypertension knowledge (%) | | | | |
| Baseline | 72.3 (70.5, 74.1) | 71.0 (69.2, 72.8) | – |  |
| 8 weeks | 73.1 (71.2, 74.9) | 73.7 (71.9, 75.5)* | -1.97 (-4.40, 0.45), .11 | |
| 16 weeks | 75.5 (73.7, 77.3)* | 75.0 (73.2, 76.8)* | -0.83 (-3.24, 1.58), .50 | |
| 24 weeks | 76.6 (74.8, 78.4)* | 76.3 (74.5, 78.1)* | -1.02 (-3.41, 1.37), .40 | |
| Self-efficacy for coping with chronic disease | | | | |
| Baseline | 7.31 (7.09, 7.53) | 6.98 (6.76, 7.21) | – | |
| 8 weeks | 7.38 (7.15, 7.61) | 7.18 (6.95, 7.40)* | -0.12 (-0.39, 0.15), .37 | |
| 16 weeks | 7.45 (7.22, 7.67) | 7.25 (7.02, 7.47)* | -0.13 (-0.39, 0.14), .36 | |
| 24 weeks | 7.49 (7.27, 7.72) | 7.25 (7.02, 7.47)* | -0.08 (-0.35, 0.18), .55 | |

*Indicates significant difference from baseline.
